# A highly conserved complete accessory *Escherichia coli* type III secretion system 2 is widespread in bloodstream isolates of the ST69 lineage

**DOI:** 10.1038/s41598-020-61026-x

**Published:** 2020-03-05

**Authors:** Stephen Fox, Cosmika Goswami, Matthew Holden, James P. R. Connolly, James Mordue, Nicky O’Boyle, Andrew Roe, Martin Connor, Alistair Leanord, Tom J. Evans

**Affiliations:** 1 Institute of Infection, Immunity and Inflammation, University of Glasgow, Glasgow, UK; 2 School of Medicine, University of St. Andrews, St. Andrews, UK; 3 Dumfries and Galloway Royal Infirmary, Dumfries, UK

**Keywords:** Bacterial genetics, Genetics research

## Abstract

Bacterial type III secretion systems (T3SSs) play an important role in pathogenesis of Gram-negative infections. Enteropathogenic and enterohemorrhagic *Escherichia coli* contain a well-defined T3SS but in addition a second T3SS termed *E. coli* T3SS 2 (ETT2) has been described in a number of strains of *E. coli*. The majority of pathogenic *E. coli* contain elements of a genetic locus encoding ETT2, but which has undergone significant mutational attrition rendering it without predicted function. Only a very few strains have been reported to contain an intact ETT2 locus. To investigate the occurrence of the ETT2 locus in strains of human pathogenic *E. coli*, we carried out genomic sequencing of 162 isolates obtained from patient blood cultures in Scotland. We found that 22 of 26 sequence type (ST) 69 isolates from this collection contained an intact ETT2 together with an associated *eip* locus which encodes putative secreted ETT2 effectors as well as *eilA*, a gene encoding a putative transcriptional regulator of ETT2 associated genes. Using a reporter gene for *eilA* activation, we defined conditions under which this gene was differentially activated. Analysis of published *E. coli* genomes with worldwide representation showed that ST69 contained an intact ETT2 in these strains as well. The conservation of the genes encoding ETT2 in human pathogenic ST69 strains strongly suggests it has importance in infection, although its exact functional role remains obscure.

## Introduction

Pathogenic bacteria possess a number of different secretion systems that facilitate host infection as well as interbacterial competition^1^. One of these is the type III secretion system (T3SS), which is found in a number of different Gram-negative pathogens and is key to the ability of these microbes to cause disease^2–4^. Broadly, T3SS comprise two elements: a highly conserved multiprotein structural complex that forms the conduit between the bacterial and the host cell; and various effector proteins that are translocated through this channel. Genes encoding the T3SS channel, or needle complex, are contained within pathogenicity islands comprised of a single cluster of genes^5,6^. Genes encoding effectors are more widely spread within the genome and vary greatly between different bacterial species.

Certain strains of *Escherichia coli* possess a well-defined T3SS, notably enteropathogenic *E. coli* (EPEC) and enterohemorrhagic *E. coli* (EHEC). This T3SS is encoded on the locus of enterocyte effacement (LEE) and in concert with its secreted effectors, produces the characteristic attaching and effacing lesions that mediate close attachment of the pathogen with the intestinal epithelial wall^7^. Whole genome sequencing of strains of EHEC revealed the presence of a putative additional T3SS^8,9^, which has been termed *E. coli* T3SS 2 (ETT2). The gene cluster encoding this additional T3SS shows significant homology to the SPI-1 T3SS of *Salmonella* serotype Typihimurium^10,11^. However, unlike the LEE, the T3SS first described in EHEC and EPEC, there do not appear to be any putative effector proteins encoded within ETT2 and there are some differences in the structural genes present as well^11^. Compared to the SPI1 T3SS of *S*. Typhimurium, the ETT2 apparently lacks homologues of genes encoding the needle tip complex, *SipBCD*. Further studies attempted to delineate the frequency with which this ETT2 locus was found in different *E. coli* strains^12–14^. However, a study by Ren *et al*.^15^ showed that although the ETT2 locus was present in many lineages of *E. coli*, it had undergone extensive mutational attrition. The phylogenetic analysis showed that ETT2 was absent in what is thought to be the oldest phylogroup of *E. coli*, B2^16,17^, which contains many uropathogenic *E. coli*, but had been acquired by the divergence of the next oldest phylogroup, D. Analysis showed multiple inactivating mutations were present within the locus, which would render the T3SS functionless, including the ETT2 locus in the EHEC O157 strains in which it was originally described. However, a complete and potentially fully functional ETT2 was found in the enteroaggregative *E. coli* O42 (EAEC O42) strain; other *E. coli* strains analysed either had no ETT2 locus, or it had undergone extensive deletion and/or mutational inactivation. Ren *et al*. also showed that *E. coli* strains with the most intact ETT2 locus also carried an additional T3SS-like island adjacent to the *selC* tRNA gene, the *eip* locus, which encoded homologues of translocated proteins from the *Salmonella* pathogenicity island I (Spi-1) T3SS, as well as genes encoding a transcriptional regulator (*eilA*), a chaperone (*eicA*) and an outer membrane invasion/intimin-like protein (*eaeX*)^15,18^.

Functional effects of ETT2 remain unclear. Mutational analysis of the ETT2 cluster in an avian pathogenic *E. coli* showed it had reduced virulence, even though the cluster had undergone mutational attrition and could not encode a functional T3SS, suggesting potential alternative roles in pathogenesis^19^. Other studies have also suggested a role for proteins encoded in the ETT2 in virulence of avian pathogenic *E. coli* and K1 strains^20–22^. A recent study examined the role of the putative transcriptional regulator gene *eilA* at the *selC* locus in EAEC strain O42^18^. This demonstrated that *eilA* was responsible for regulating transcription of genes within the *selC* locus, as well as *eivF* and *eivA* within the ETT2 locus. Mutants lacking *eilA* were less adherent to epithelial cells and had reduced biofilm formation; this phenotype was also observed for mutants in the *eaeX* gene which encodes the invasin/intimin homologue. This suggested important functional roles of the *selC* and ETT2 loci in pathogenesis of this strain of *E. coli*.

Hitherto, there is no evidence of intact ETT2 in human pathogenic strains of *E. coli* other than a few strains of EAEC. However, given the findings described above, we hypothesised that ETT2 might be of importance in human infections caused by *E. coli* phylogroups other than B2. In particular, given the roles of T3SSs in attachment, invasion and immune evasion, we hypothesised that strains with an intact ETT2 might be found within invasive bloodstream isolates of extraintestinal pathogenic *E. coli*, where the ETT2 might have allowed the organism to overcome epithelial barriers and immune clearance. Thus, we set out to determine whether an intact ETT2 was present in a collection of invasive bloodstream isolates of *E. coli*. We have studied 162 isolates of *E. coli* isolated from bacteremic patients in Scotland from 2013 and 2015, which we have subjected to whole genome sequencing. Within this group, we identified 26 strains of *E. coli* sequence type (ST) 69, of phylogroup D, which were largely derived from community-acquired sources. Virtually all of these strains had a completely intact ETT2 and *selC* locus, with no inactivating mutations. Similarly, intact ETT2/*selC* loci were also found in some minor ST types in our collection. The *eilA* transcriptional regulator was functional in these strains. Analysis of *E. coli* strains with worldwide representation also showed that ST69 contained an intact ETT2 in these strains as well. Our results show that an intact ETT2 locus is widely present in human pathogenic *E. coli* ST69 strains, suggesting a functional role for this cryptic T3SS in human disease caused by this sequence type.

## Results

### ETT2 locus within Scottish *E. coli* blood stream isolates

**Figure 1:**
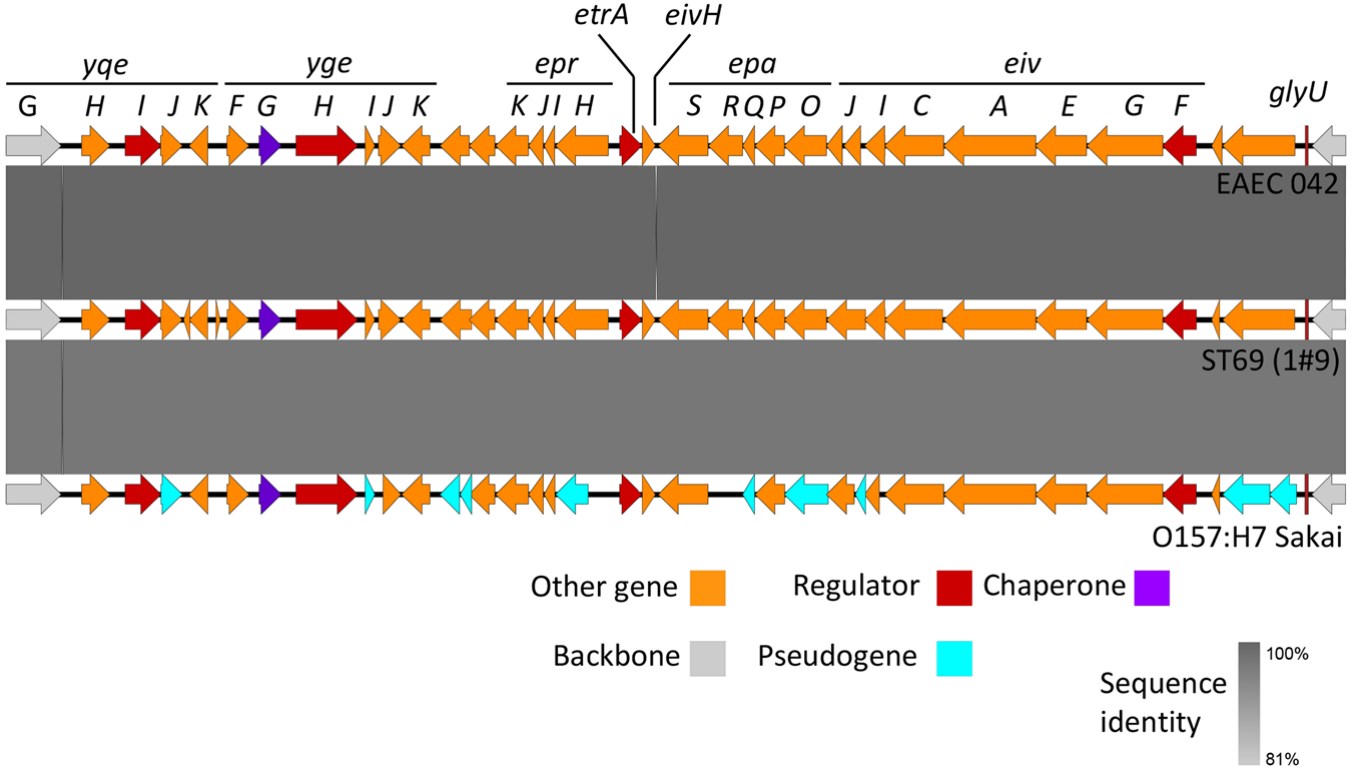
Comparisons of the ETT2 locus between EAEC 042, ST69 (1#9) and O157:H7 Sakai. Degree of identity is shown by the level of grey shading as indicated. Genes are colour coded according to putative function as shown.

We have performed whole genome sequencing and analysis of 162 isolates of *Escherichia coli* obtained from blood cultures of patients within Scotland in 2013 and 2015^23^. Sequence comparisons with other isolates of *E. coli* showed that strains belonging to ST69 contained an intact ETT2 locus. The gene content of this locus from one of these ST69 strains, ST69 1#9, was compared to the complete ETT2 found in enteroaggregative *E. coli* strain 042 (EAEC 042) and the degenerate ETT2 found in *E. coli* O157:H7 Sakai (Fig. 1). An ETT2 locus in this ST69 strain was found in the ~30 kb region spanning the *yqeG* gene and the tRNA gene *gluU* with over 98% identity to the ETT2 locus in EAEC 042. Importantly, this locus did not contain any of the deletions, insertions or inactivating mutations found in the *E. coli* O157:H7 Sakai strain and thus was characterised as intact.

**Figure 2:**
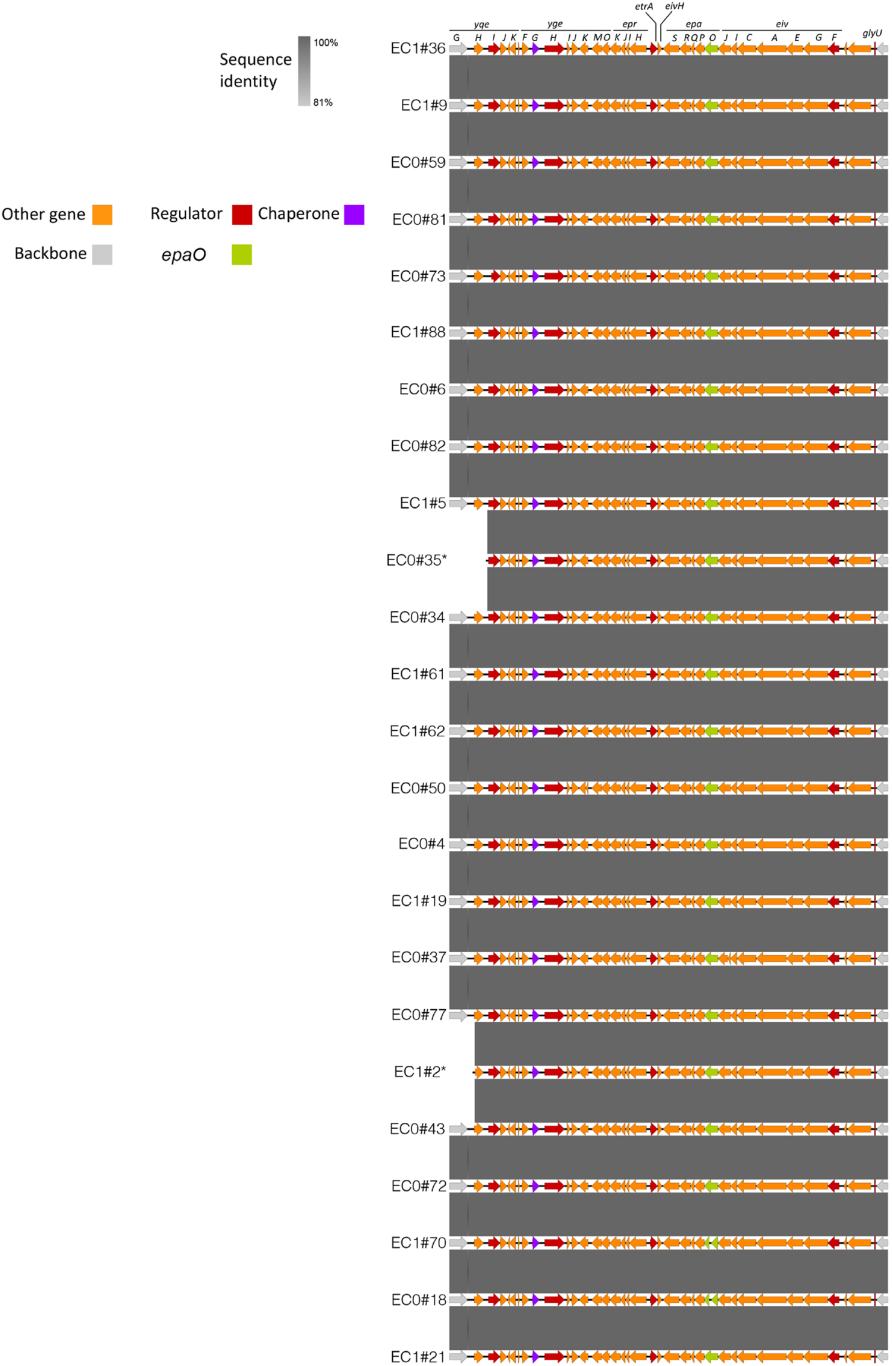
Comparison of the ETT2 operon in 24 ST69 strains. Degree of identity is shown by the level of grey shading as indicated. Genes are colour coded according to putative function as shown. The *epaO* gene is shown green.

We extended this analysis to compare all of the ST69 strains in our collection over this region. Of 26 ST69 genomes sequenced, 24 were assembled in one contig covering this region, shown compared to each other in Fig. 2. In all these assemblies, there was a greater than 95% identity between the sequences and the reference genome of the ETT2 in EAEC 042 (Table 1). Two strains appeared to lack the extreme left-hand end of the complete ETT2 locus (ECO#35 and EC1#2), and two strains had a stop codon in the *epaO* gene at the same site as noted for *E. coli* O157:H7 Sakai (EC1#70 and ECO1#18; gene highlighted in green); no other ST69 strains had any inactivating mutations.

**Table 1:** Similarities of length and identity between the ETT2 and *eip* loci in the strains indicated.

ST69 Samples	ST Type	ETT2				*eip*			
		Hits	Identity	Length	Bit score	Hits	Identity	Length	Bit score
EC0_4	ST69	1	98.9%	27163	48483	34	95.3%	13862	21987
EC0_59	ST69	1	98.9%	27163	48483	34	95.3%	13862	21987
EC0_81	ST69	1	98.9%	27163	48483	34	95.3%	13862	21987
EC0_82	ST69	1	98.9%	27163	48483	34	95.3%	13862	21987
EC1_19	ST69	1	98.9%	27163	48483	34	95.3%	13862	21987
EC1_36	ST69	1	98.9%	27163	48483	34	95.3%	13862	21987
EC1_9	ST69	1	98.9%	27163	48483	34	95.3%	13862	21987
EC0_50	ST69	1	98.9%	27163	48477	34	95.3%	13862	21987
EC0_77	ST69	1	98.9%	27163	48483	34	95.3%	13862	21981
EC1_5	ST69	1	98.9%	27163	48483	34	95.3%	13862	21981
EC1_61	ST69	1	98.9%	27163	48477	34	95.3%	13862	21987
EC0_6	ST69	1	98.9%	27163	48483	34	95.3%	13862	21976
EC1_88	ST69	1	98.9%	27163	48483	34	95.3%	13862	21976
EC0_34	ST69	1	98.9%	27163	48471	34	95.3%	13862	21987
EC0_18	ST69	1	98.9%	27163	48466	34	95.3%	13862	21987
EC0_72	ST69	1	98.9%	27163	48466	34	95.3%	13862	21987
EC1_21	ST69	1	98.9%	27163	48466	34	95.3%	13862	21987
EC1_70	ST69	1	98.9%	27163	48466	34	95.3%	13862	21987
EC0_43	ST69	1	98.9%	27164	48468	34	95.3%	13862	21976
EC0_73	ST69	1	98.9%	27173	48436	34	95.3%	13862	21987
EC1_2	ST69	2	98.9%	27130	48392	34	95.3%	13862	21981
EC0_37	ST69	1	98.9%	27163	48471	35	95.3%	13812	21872
EC1_62	ST69	1	98.9%	27163	48471	33	95.5%	13573	21638
EC0_35	ST69	1	98.9%	26248	46793	35	94.9%	12839	20092

Conservation was determined using BLAST against the EAEC 042 reference.

**Figure 3:**
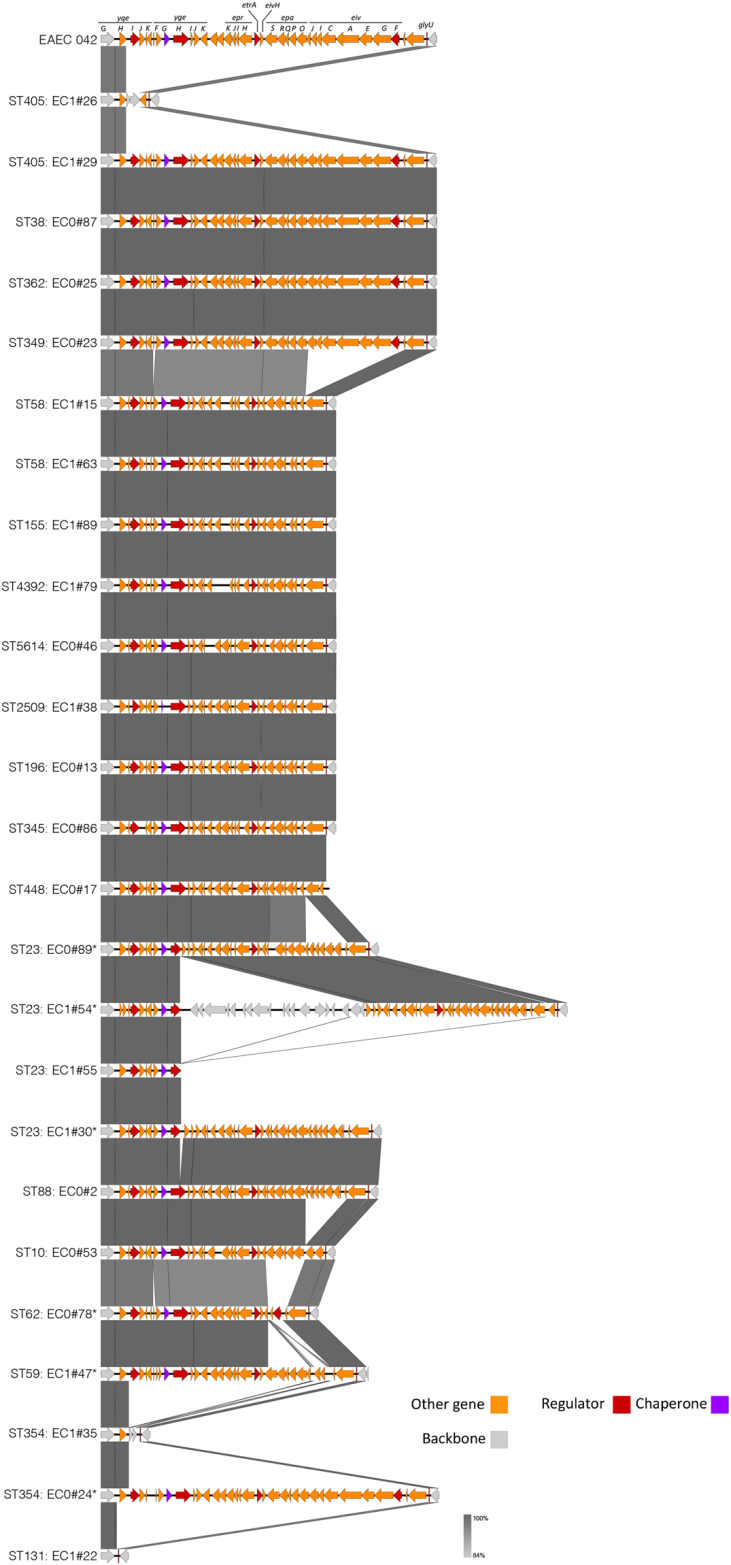
Comparison of elements of the ETT2 locus found in non-ST69 strains. Degree of identity is shown by the level of grey shading as indicated. Genes are colour coded according to putative function as shown. Genes unrelated to the ETT2 locus genes are coloured grey.

Next, we analysed other STs within our collection of bacteremic isolates for the presence of the ETT2 locus (Fig. 3). 4 non-ST69 isolates contained an intact ETT2 region, belonging to ST405, 38, 362 and 349. BLAST percentage identity and length coverage of the ETT2 from these strains to EAEC 042 is shown in Table 2; all are closely related to ST69 (Supplementary Fig. S1). Other strains showed variable loss and/or degradation of the locus as previously described. Notably, none of the common epidemic strain ST131 (phylogroup B2) contains any elements of this ETT2 region – one representative example is shown at the bottom of Fig. 3.

**Table 2:** Similarities of the ETT2 locus between the strains indicated.

Sample	Strain type	Hits	Identity	Length	Bit score
EC0_23	ST349	1	98.9%	27161	48447
EC0_87	ST38	1	98.8%	27160	48268
EC1_29	ST405	1	98.7%	27161	48220
EC0_25	ST362	1	98.7%	27161	48172

Conservation was determined using BLAST against the EAEC 042 reference.

### selC/eip locus within Scottish blood culture isolates

**Figure 4:**
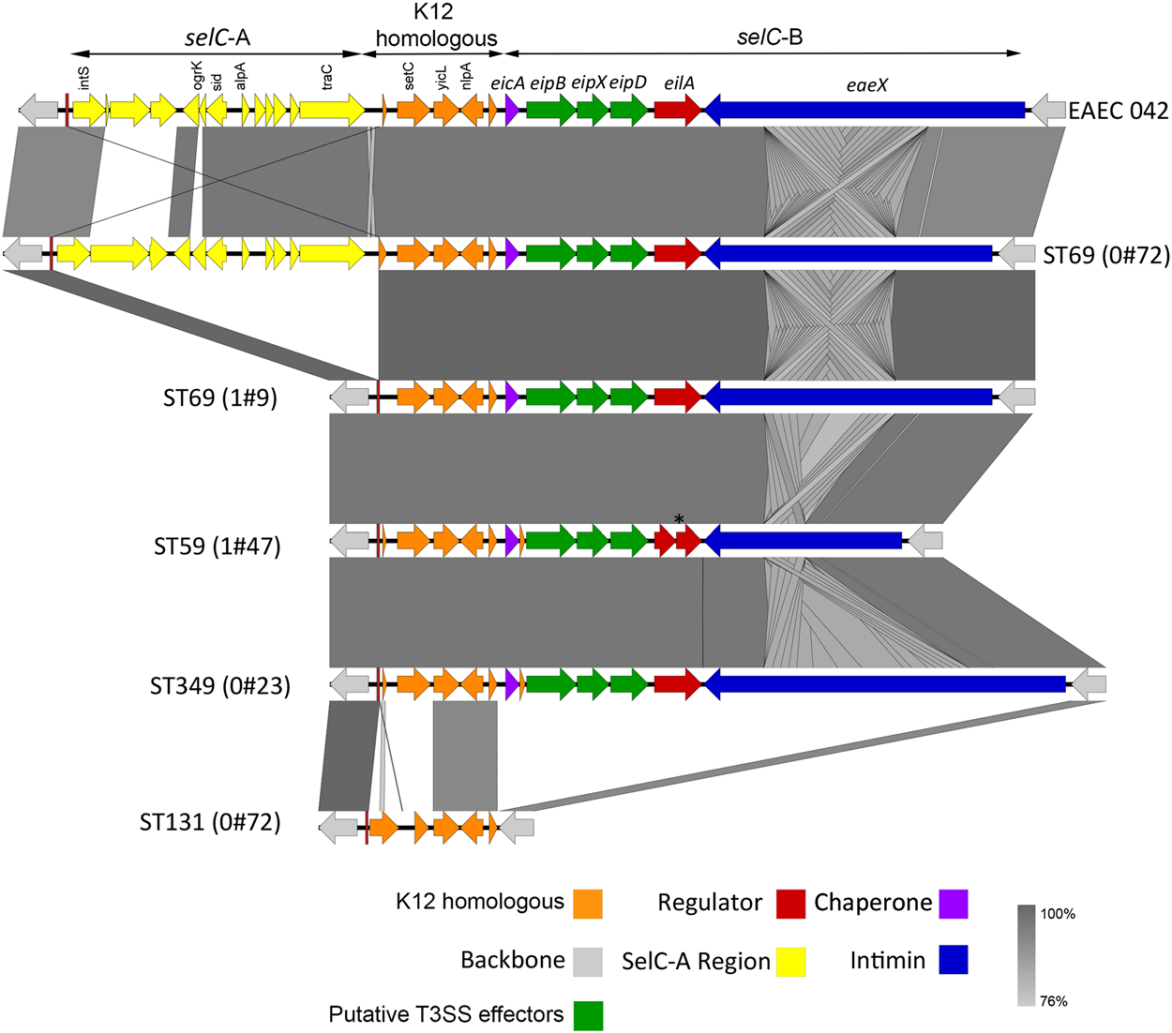
Comparison of the *selC* operon in different strains. Degree of identity is shown by the level of grey shading as indicated. Genes are colour coded according to putative function as shown. *Shows the position of a frameshift mutation in the *eilA* gene of sample 1#47 (ST59).

Closely associated with an intact ETT2 region is a group of genes related to type III secretion effectors adjacent to the *selC* tRNA gene^15,18^. Two distinct genome insertions were noted at this site: *selC-*A and *selC-*B, as defined and described by Sheikh *et al*.^18^. *selC-*A contains mainly phage related genes. *selC*-B contains homologues of putative type III secretion effectors (*eipB, eipX* and *eipD*), a putative type III effector chaperone, *eicA*, a transcriptional regulator *eilA*, and a gene *eaeX*, which encodes a large protein containing bacterial immunoglobulin repeats with homology to outer membrane adhesion/invasion protein intimin found in *Yersinia* spp. as well as intimins of invasive *E. coli* strains. Comparison of this region with representative ST69 and other strains compared to EAEC 042 is shown in Fig. 4. In EAEC 042 *selC-*A lies between an intact copy of the *selC* gene and a 21 bp direct repeat of the 3′ end of the *selC* tRNA gene. Three backbone genes then intervene (*setC, yicL, nlpA*) before the region of the *selC-*B region. All ST69 strains in our isolates contained the *selC-*B locus with over 95% identity to the EAEC 042 region (Table 1). The variations were found within the central domain of the EaeX product, which contains the bacterial immunoglobulin (Big) repeats, with variation in the number of repeats contained within this domain. A similar region was also found in non-ST69 isolates; one ST59 strain and one ST349 strain also possessed the ETT2 locus. These major differences in the number of Big repeats between the strains is shown in Supplementary Fig. S2. Domain analysis with ScanProsite also identified an N terminal LYSM domain, a module that recognizes polysaccharides containing N-acetylglucosamine (GlcNAc) residues including peptidoglycan^24^. The Big repeat number was conserved within the ST69 strains suggesting that once the *eaeX* gene was acquired within this strain it has been maintained; there are too few isolates with the *eaeX* gene from other STs to be able to comment on its conservation or otherwise in these groups. As with the ETT2 locus, the *selC-*B region was entirely missing in ST131 isolates. The *selC-*A region was largely absent from our isolates but was partially present in one of the ST69 isolates (ECO#72, Fig. 4).

**Figure 5:**
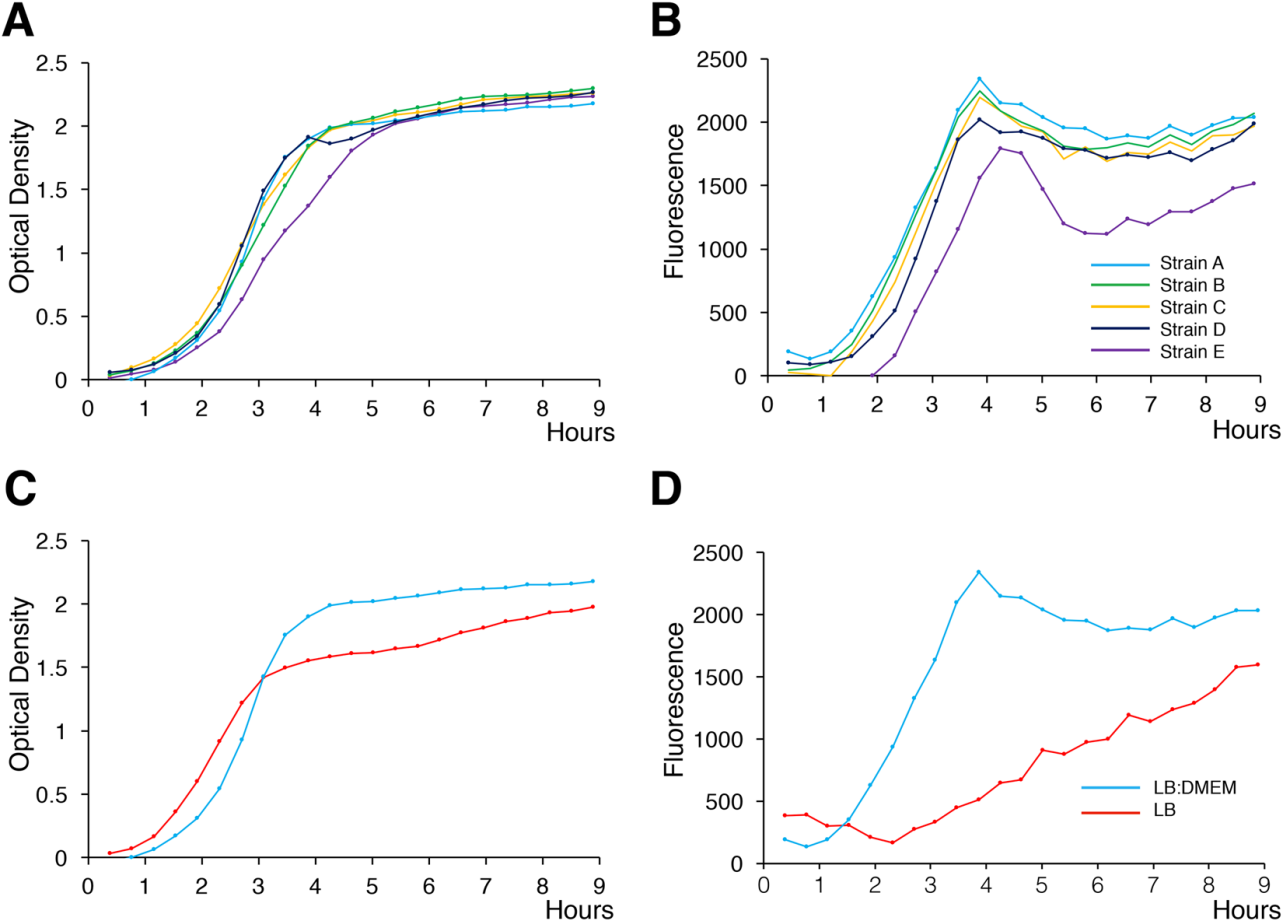
Activity of the eilA reporter in different strains and media. (**A**,**B**) Graphs show growth (Optical Density, panels A) and reporter activity (GFP fluorescence, panels B) at the times indicated. The strains are: EC1#2 (**A**), EC1#19 (**B**), EC1#5 (**C**), EC1#21 (**D**), and EC1#9 (**E**), all grown in LB:DMEM mixture. Each point is the mean of a triplicate determination; error bars (sem) are contained within the points. (**C,D**) strain EC1#2 is grown in the different media as indicated.

*EilA* has been shown to regulate genes within the *selC*-B region as well as the ETT2 island adjacent to the tRNA *glyU* gene^18^. We wished to determine if we could define conditions under which *eilA* was transcriptionally active, and hence activating the ETT2 island. We constructed a reporter gene containing 500 bp of upstream sequence from the *eilA* gene found in the neonatal meningitis associated *E. coli* strain CE10^25^. Analysis of this region in strain EC1#2 used for the detailed reporter expression studies showed 96.4% identity with the same region in CE10 and with perfect conservation of putative binding sites for *purR, fnr, argr2, argR* and a 7/8 nucleotide match to a putative site for *rpoS17*. Using this reporter in 5 of our ST69 isolates containing the ETT2 locus, we could readily detect reporter gene activity that peaked in the late log phase of growth in equal parts LB and Dulbecco’s Modified Eagle’s Medium (LB:DMEM media) (Fig. 5A,B). Previous studies of transcriptional activation of the LEE have shown this is maximal in less rich media designed for growth of eukaryotic cells such as DMEM compared to the rich medium LB^26,27^. Following optimization of growth in different media, we compared transcriptional activity of the *eilA* reporter construct in an ST69 strain grown in LB alone compared to the 1: 1 mixture of LB and DMEM (Fig. 5C,D). Growth in the different media was not significantly different but induction of the promoter was much more marked in the LB:DMEM mix. Transcription of *eilA* and two other putatively co-regulated genes was confirmed using qPCR at one time point; however, detection was at the limits of detectability and there was no significant difference between transcript levels in bacteria grown in LB or LB:DMEM (Supplementary Fig. S3). Given the short half-life of bacterial mRNAs of the order of 2–10 minutes^28^, we feel the reporter assay is a more sensitive and accurate measurement of *eilA* promoter activity. In an attempt to identify proteins potentially secreted into the growth media by ETT2, we compared the pattern of secreted proteins from an ST69 strain with intact ETT2 between the two different media but we did not identify any putative T3SS secreted proteins or secreted components of the T3SS structural domains (data not shown).

### Presence of ETT2 and selC/eip locus within worldwide collection of *E. coli*

**Figure 6:**
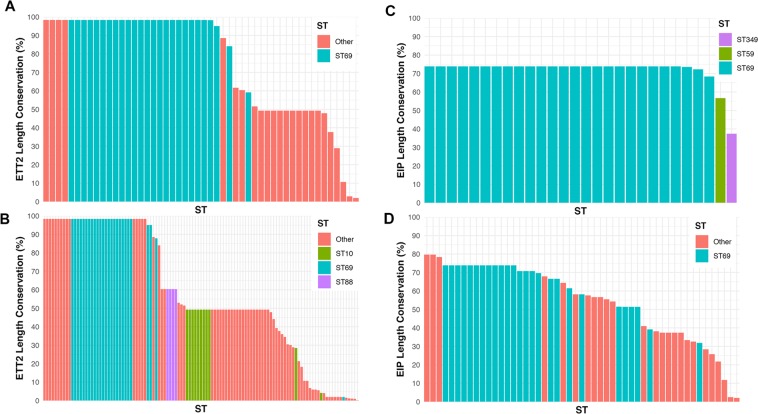
Length conservation of the ETT and *selC*/Eip locus in different strains of *E. coli* compared to the reference strain, EAEC 042. Data from the local Scottish strains (panels A and C) and global data (panels B and D) are shown. (**A**,**B**) are the comparisons for the ETT2 locus and (**C**,**D**) are for the *selC*/Eip locus. STs with fewer than 4 representatives are classed as Other in panels A, B, and D.

In order to ascertain whether the intact ETT2 and *selC*/Eip loci within ST69 strains was specific to Scotland or more widespread, we analysed the genomes of *E. coli* available from public depositories with worldwide representation. We identified 269 strains with full sequence data (Supplementary Table S1). The distribution of STs within this group compared to those within the Scottish blood culture isolates is shown in Supplementary Fig. S4. The major STs within both groups are very similar: ST131, ST69, ST73, ST95, ST12 and ST127. Analysis of the length conservation of the ETT2 locus within these sequences is shown in Fig. 6 for both the local and the global sequences. Of 26 ST69 sequences within the global collection (Fig. 6B), 22 had a 98.4% length identity to the reference ETT2 locus in the EAEC 042 strain, two strains had a 95.1% match, and one had a 87.9% match; one ST69 strain had virtually deleted the locus (2.0% length identity). Of the non-ST69 strains that showed >95% conservation of the ETT2 locus, there was no ST present with more than 4 members. Two ST38 strains were included in this group, also found within our collection of Scottish bacteremic strains with high conservation of the ETT2 locus. The length conservation of the *selC*/Eip locus (over the *selC*-B region) for the local and global *E. coli* strains is shown in Fig. 6C,D. 19/26 (73%) of the ST69 strains had a >60% length conservation compared to the reference EAEC 042 strain. The major differences in length of the different strains from the EAEC reference were in the *eaeX* gene, which contain different numbers of the bacterial immunoglobulin-like repeats. As for the ETT2 locus, the ST131 strains did not contain any of the *selC*-B locus either.

## Discussion

We report here the presence of genomic regions encoding ETT2 and associated putative T3SS effectors within *E. coli* ST69 isolates from bacteremic patients within Scotland. In virtually all of the isolates, the two regions encoding these proteins contained a full complement of genes with no deletion, insertions or inactivating mutations suggesting that the ETT2 and associated effectors could be functionally active. This is in contrast to the vast majority of ETT2 sequences reported to date, which have undergone significant mutational attrition. The conserved nature of the ETT2 sequences reported here strongly suggests that there has been selection pressure for these regions to be conserved within the ST69 lineage.

ST69 belongs to phylogroup D of the *E. coli* lineage. We did not detect ETT2 in *E. coli* of ST131, which is phylogroup B2. Although not completely clear, our data are in agreement with the origin of the different phylogroups as discussed by Ren *et al*.^15^, who suggest that ETT2 is not present in the ancestral B2 phylogroup but was acquired at some point in the evolution of the D group. Feature free profiling also supports the view that B2 is the ancestral group, with phylogroup D diverging thereafter^16^. Subsequent lineages show significant mutational attrition of the ETT2 locus, although our data show strong conservation in the isolates of ST69 studied here. ST69 is one of the common STs found in bloodstream isolates of *E. coli*. In our collection, ST69 was mostly found in infections acquired from the community^23^. The natural environment of these human pathogenic *E. coli* is the gastrointestinal tract; passage into blood is predominantly through ascending infection into the bladder and renal tract. Evolutionary pressure to retain ETT2 might therefore have arisen through its ability to provide a selective advantage in gut colonization and/or in infection of the renal tract. Importantly, we also found highly significant conservation of the ETT2 and *selC*/Eip loci in *E. coli* strains from global collections, showing that the preservation of these regions is not confined to local Scottish strains.

We noted that two strains had a stop codon in the *epaO* gene at the same site as noted for *E. coli* O157:H7 Sakai (EC1#70 and ECO1#18). *epaO* is homologous to the *Salmonella* Typhimurium T3SS gene, *spaO*^13^, which encodes a protein that forms part of the cytoplasmic sorting platform essential for energizing and sorting substrates for delivery to the needle complex^29^. *spaO* is essential for type III secretion in *S*. Typhimurium^30^. Recent work has shown that *spaO* produces two protein products by tandem translation: a full-length protein and a shorter C terminal portion that is translated from an internal ribosome binding site and alternative initiator codon^31^. Both are needed for functionality of the T3SS in *S*. Typhimurium, so the loss of the full-length product of *epaO* will likely also render the ETT2 non-functional.

The functional effects of ETT2 remain obscure. In strains with a disrupted ETT2, genetic deletion does seem to confer a changed phenotype, with defective invasion and survival within brain microvascular endothelial cells^22^; this suggests even these apparently non-functional regions have a pathogenic role or can be complemented by other gene products. Additionally, experiments in avian strains with ETT2 also suggest a functional role for the ETT2 in pathogenesis^20^. ETT2 has also been implicated in the control of gene expression from the locus of enterocyte effacement in enterohemorrhagic *E. coli* O157^32^. We could not identify any putative secreted ETT2 substrates from the ST69 strains reported here. A recent study of *E. coli* serotype O2 that causes avian coccobacillosis also failed to identify potential ETT2 secreted proteins, but did find that the intact ETT2 mediated expression and secretion of flagellar proteins, as well as other changes in cell surface behaviour^33^. It may be that the conditions under which the ETT2 mediates secretion have not been identified, or that it carries out different functions.

In summary therefore, we show here that the ST69 strain of human pathogenic *E. coli* has an intact genetic locus for ETT2 and associated proteins. The preservation of these sequences in the ST69 strain suggest that its functional effects might confer a significant selection advantage. However, its exact functional effects remain obscure.

## Methods

### Sequencing and genome analysis

Whole genome sequencing of 162 strains of *E. coli* from human clinical samples were collected and sequenced as previously described^23^. Mean Phred score of the reads was 34.57 (99.9% base call accuracy), mean N50 of the assemblies was 355, 277, and the mean number of contigs assembled per sample was 62.6. The data for the individual samples is shown in Supplementary Table S2.

For pangenome analysis, Illumina reads were assembled using the de novo assembler SPAdes^34^. After filtering contigs less than 100 bp long, genomes were annotated for genus *Escherichia* using PROKKA^35^ with default parameters. Annotated genomes were then studied using the pan-genome pipeline Roary^36^ using minimum blastp identity as 95% and percentage of isolates to be in the core genome taken as 99%. The presence and absence of genes in accessory genome (>5% and <99% isolates) was used to create the binary tree. The ETT2 locus genes were identified using BLAST against the ETT2 region of EAEC 042 strain.

Comparison between selected sequences were made and visualised using Easyfig^37^. MLST typing was performed *in silico* using the Achtman profile in BIGSdb^38^.

Identification of the ETT2 and *eip* loci was performed using BLAST. Coverage and sequence percentage identities to the reference genome of EAEC 042 are shown in Table 1. The ETT2 locus was termed intact if it contained no deletion, insertions or inactivating mutations.

Maximum Likelihood trees of the sequences shown in Fig. S1 was performed using RaxML^39^ on core genes (2804 genes) using a Generalised Time Reversible Gamma model (-m set to GTRGAMMA), the algorithm set to rapid Bootstrap analysis and search for bestscoring ML tree in one program with flag -f set to a, number of bootstraps set to 100, and the seed values in flags -p and -x set to 12345.

Global representative sequences filtered as bloodstream isolates were downloaded from the EnteroBase archive^40^ with accession numbers set out in Table S1. They were from diverse geographical locations and patients as indicated in available metadata.

### Domain analysis of the EaeX protein

Domain identification within the EaeX protein was determined using ScanProsite and the PROSITE data base^41^.

### Growth and eilA reporter assay

Growth media used in this study were DMEM (Invitrogen, UK), LB, and a 1:1 mix of LB with DMEM. The *eilA* reporter construct contains a ~500 bp fragment upstream of the *eilA* promoter from the CE10 strain of *E. coli* (Accession number NC017646) that was cloned into a plasmid (pAJR70) used in a previous study for the assessment of transcription of ETT1 operons by enhanced green fluorescent protein (GFP) monitoring from liquid culture^42^. The different bacterial strains were transformed with this plasmid using standard methods. Chloramphenicol (25 µg/ml) was added to media when required for the selection of strains containing the eilA reporter. Induction of GFP in the different media at 37 °C was measured using a fluorescence plate-reader (FLUOstar Optima; BMG; Labtech, UK). Optical densities and fluorescence were recorded every 24 minutes for 9 hours. Measurements from bacteria transformed with the promoterless pAJR70 showed there was no signal produced above that of the fluorescence of bacteria alone which was subtracted from all readings.

### Type III secretion assay

Secreted proteins were extracted by trichloroacetic acid precipitation performed as previously described^43^. Briefly, overnight LB cultures were diluted 1/100 in 50 ml of the culture media and grown for 9 hours before precipitation of secreted proteins. Secreted proteins were resuspended in 150 µl of loading buffer and analysed by SDS-PAGE.

### Quantitative PCR

Bacteria were grown to late-log phase in the indicated media and harvested into RNAprotect (Qiagen) according to the manufacturer’s guidelines. RNA was extracted using a RNAeasy kit (Qiagen) according to the manufacturer’s guidelines. Contaminating DNA was removed using Turbo DNAse (Ambion) followed by phenol-chloroform extraction and ethanol precipitation. RNA was reverse transcribed and quantitative PCR performed using a Syber Green PowerUp master mix (Applied Biosystems) according to the manufacturer’s protocol. Specific primers used are shown in Supplementary Table S3. Amplification was performed using a 7500 series RT PCR system (Applied Biosystems). Quantification of results was performed using the ΔΔCt method of Livak and Schmittgen, using *gapA* as a reference gene^44^. Results were expressed as fold induction in LB:DMEM relative to the level in LB alone.

### Ethical approval

Advice was sought from the Local Research Ethics Committee of Greater Glasgow and Clyde NHS Board. Specific ethical permission was deemed not to be required as the study was viewed as service improvement. Approval for access to clinical patient data was given by the Caldicott Guardian of the relevant health boards, who is the designated regulator of confidential patient information within NHS Scotland.

## Supplementary information

Supplementary Information.

Table S1.

Table S2.

## Data Availability

Illumina sequences are deposited in the European Nucleotide Archive (ENA: www.ebi.ac.uk/ena) under project PRJEB12513. The global *E. coli* accession numbers from the ENA are set out in Table S1.

## References

[1] Green, E. R. & Mecsas, J. Bacterial Secretion Systems: An Overview. *Microbiol Spectr* **4**, 10.1128/microbiolspec.VMBF-0012-2015 (2016). 10.1128/microbiolspec.VMBF-0012-2015 PMC4804464 26999395

[2] Cornelis GR (2006). The type III secretion injectisome. Nature reviews. Microbiology.

[3] Mota LJ, Cornelis GR (2005). The bacterial injection kit: type III secretion systems. Ann. Med..

[4] Deng W (2017). Assembly, structure, function and regulation of type III secretion systems. Nature reviews. Microbiology.

[5] Hueck CJ (1998). Type III protein secretion systems in bacterial pathogens of animals and plants. Microbiol. Mol. Biol. Rev..

[6] Gaytán, M. O., Martínez-Santos, V. I., Soto, E. & González-Pedrajo, B. *Type Three Secretion System in Attaching and Effacing Pathogens*. **6**, 10.3389/fcimb.2016.00129 (2016). 10.3389/fcimb.2016.00129 PMC5073101 27818950

[7] Stevens, M. P. & Frankel, G. M. The Locus of Enterocyte Effacement and Associated Virulence Factors of Enterohemorrhagic Escherichia coli. *Microbiol Spectr* **2**, EHEC-0007-2013, 10.1128/microbiolspec.EHEC-0007-2013 (2014). 10.1128/microbiolspec.EHEC-0007-2013 26104209

[8] Hayashi T (2001). Complete genome sequence of enterohemorrhagic Escherichia coli O157:H7 and genomic comparison with a laboratory strain K-12. DNA Res..

[9] Perna NT (2001). Genome sequence of enterohaemorrhagic Escherichia coli O157:H7. Nature.

[10] Lostroh CP, Lee CA (2001). The Salmonella pathogenicity island-1 type III secretion system. Microbes and infection/Institut Pasteur.

[11] Zhou M, Guo Z, Duan Q, Hardwidge PR, Zhu G (2014). Escherichia coli type III secretion system 2: a new kind of T3SS?. Vet. Res..

[12] Hartleib S, Prager R, Hedenstrom I, Lofdahl S, Tschape H (2003). Prevalence of the new, SPI1-like, pathogenicity island ETT2 among Escherichia coli. Int. J. Med. Microbiol..

[13] Makino S (2003). Distribution of the secondary type III secretion system locus found in enterohemorrhagic Escherichia coli O157:H7 isolates among Shiga toxin-producing E. coli strains. J. Clin. Microbiol..

[14] Miyazaki J, Ba-Thein W, Kumao T, Akaza H, Hayashi H (2002). Identification of a type III secretion system in uropathogenic Escherichia coli. FEMS Microbiol. Lett..

[15] Ren CP (2004). The ETT2 gene cluster, encoding a second type III secretion system from Escherichia coli, is present in the majority of strains but has undergone widespread mutational attrition. J. Bacteriol..

[16] Sims GE, Kim SH (2011). Whole-genome phylogeny of Escherichia coli/Shigella group by feature frequency profiles (FFPs). Proc. Natl. Acad. Sci. USA.

[17] Lecointre G, Rachdi L, Darlu P, Denamur E (1998). Escherichia coli molecular phylogeny using the incongruence length difference test. Mol. Biol. Evol..

[18] Sheikh J (2006). EilA, a HilA-like regulator in enteroaggregative Escherichia coli. Mol. Microbiol..

[19] Ideses D (2005). A degenerate type III secretion system from septicemic Escherichia coli contributes to pathogenesis. J. Bacteriol..

[20] Wang S (2017). Escherichia coli type III secretion system 2 regulator EtrA promotes virulence of avian pathogenic Escherichia coli. Microbiology.

[21] Wang S (2016). Escherichia coli Type III Secretion System 2 ATPase EivC Is Involved in the Motility and Virulence of Avian Pathogenic Escherichia coli. Front Microbiol.

[22] Yao Y (2009). The type III secretion system is involved in the invasion and intracellular survival of Escherichia coli K1 in human brain microvascular endothelial cells. FEMS Microbiol. Lett..

[23] Goswami, C. *et al*. Genetic analysis of invasive Escherichia coli in Scotland reveals determinants of healthcare-associated versus community-acquired infections. *Microb Genom* **4**, 10.1099/mgen.0.000190 (2018). 10.1099/mgen.0.000190 PMC6096937 29932391

[24] Buist G, Steen A, Kok J, Kuipers OP (2008). LysM, a widely distributed protein motif for binding to (peptido)glycans..

[25] Lu S (2011). Complete genome sequence of the neonatal-meningitis-associated Escherichia coli strain CE10. J. Bacteriol..

[26] Puente JL, Bieber D, Ramer SW, Murray W, Schoolnik GK (1996). The bundle-forming pili of enteropathogenic Escherichia coli: transcriptional regulation by environmental signals. Mol. Microbiol..

[27] Leverton LQ, Kaper JB (2005). Temporal expression of enteropathogenic Escherichia coli virulence genes in an *in vitro* model of infection. Infect. Immun..

[28] Dutta T, Srivastava S (2018). Small RNA-mediated regulation in bacteria: A growing palette of diverse mechanisms. Gene.

[29] Lara-Tejero M, Kato J, Wagner S, Liu X, Galan JE (2011). A sorting platform determines the order of protein secretion in bacterial type III systems. Science.

[30] Collazo CM, Galan JE (1996). Requirement for exported proteins in secretion through the invasion-associated type III system of Salmonella typhimurium. Infect. Immun..

[31] Song M (2017). Control of type III protein secretion using a minimal genetic system. Nature communications.

[32] Zhang L (2004). Regulators encoded in the Escherichia coli type III secretion system 2 gene cluster influence expression of genes within the locus for enterocyte effacement in enterohemorrhagic E. coli O157:H7. Infect. Immun..

[33] Shulman, A. *et al*. The Escherichia coli Type III Secretion System 2 Has a Global Effect on Cell Surface. mBio **9**, 10.1128/mBio.01070-18 (2018). 10.1128/mBio.01070-18 PMC6030553 29970469

[34] Bankevich A (2012). SPAdes: a new genome assembly algorithm and its applications to single-cell sequencing. J. Comput. Biol..

[35] Seemann T (2014). Prokka: rapid prokaryotic genome annotation. Bioinformatics.

[36] Page AJ (2015). Roary: rapid large-scale prokaryote pan genome analysis. Bioinformatics.

[37] Sullivan MJ, Petty NK, Beatson SA (2011). Easyfig: a genome comparison visualizer. Bioinformatics.

[38] Jolley KA, Maiden MC (2010). BIGSdb: Scalable analysis of bacterial genome variation at the population level. BMC Bioinformatics.

[39] Stamatakis A (2015). Using RAxML to Infer Phylogenies. Curr Protoc Bioinformatics.

[40] Zhou, Z. *et al*. The EnteroBase user’s guide, with case studies on Salmonella transmissions, Yersinia pestis phylogeny and Escherichia core genomic diversity. *Genome Res*., 10.1101/gr.251678.119 (2019). 10.1101/gr.251678.119 PMC6961584 31809257

[41] Sigrist CJ (2013). New and continuing developments at PROSITE. Nucleic Acids Res.

[42] Roe AJ (2003). Heterogeneous surface expression of EspA translocon filaments by Escherichia coli O157:H7 is controlled at the posttranscriptional level. Infect Immun.

[43] Tree JJ (2011). Transcriptional regulators of the GAD acid stress island are carried by effector protein-encoding prophages and indirectly control type III secretion in enterohemorrhagic Escherichia coli O157:H7. Mol Microbiol.

[44] Livak KJ, Schmittgen TD (2001). Analysis of relative gene expression data using real-time quantitative PCR and the 2(-Delta Delta C(T)) Method. Methods.

