# Supplementary material for: A highly conserved complete accessory Escherichia coli type III secretion system 2 is widespread in bloodstream isolates of the ST69 lineage
Source: Sci Rep. 2020 Mar 5;10:4135. doi: 10.1038/s41598-020-61026-x (PMC7058095; doi:10.1038/s41598-020-61026-x)
Supplement: Supplementary file 1 — Supplementary Information. [file 41598_2020_61026_MOESM1_ESM.pdf]

A highly conserved complete accessory *Escherichia coli* type III secretion system 2 is widespread in bloodstream isolates of the ST69 lineage

Stephen Fox [a], Cosmika Goswami [a], Matthew Holden [b], James P.R. Connolly [a], James Mordue [a], Nicky O'Boyle [a], Andrew Roe [a], Martin Connor [c], Alistair Leanord [a], and Tom J. Evans [a*]

[a] Institute of Infection, Immunity and Inflammation, University of Glasgow, Glasgow, UK

[b] School of Medicine, University of St. Andrews, UK

[c] Dumfries and Galloway Royal Infirmary, Dumfries, UK

[*] Corresponding author: tom.evans@glasgow.ac.uk

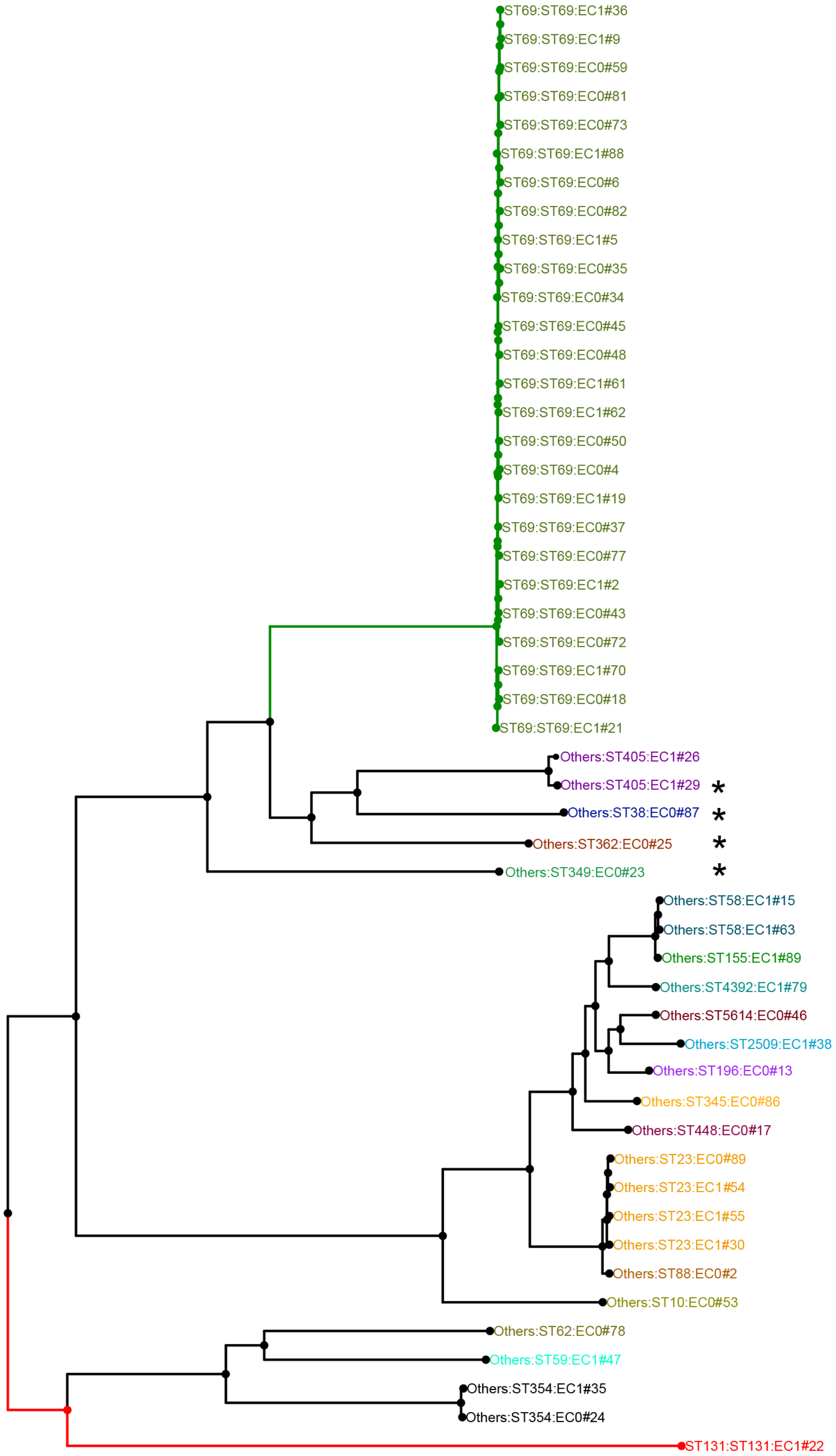

Figure S1. Maximum likelihood phylogenetic tree of the strains shown in Figure 3. Strains are colour coded according to their ST as shown. The 4 non-ST69 strains with an intact ETT2 operon are indicated by an asterisk.

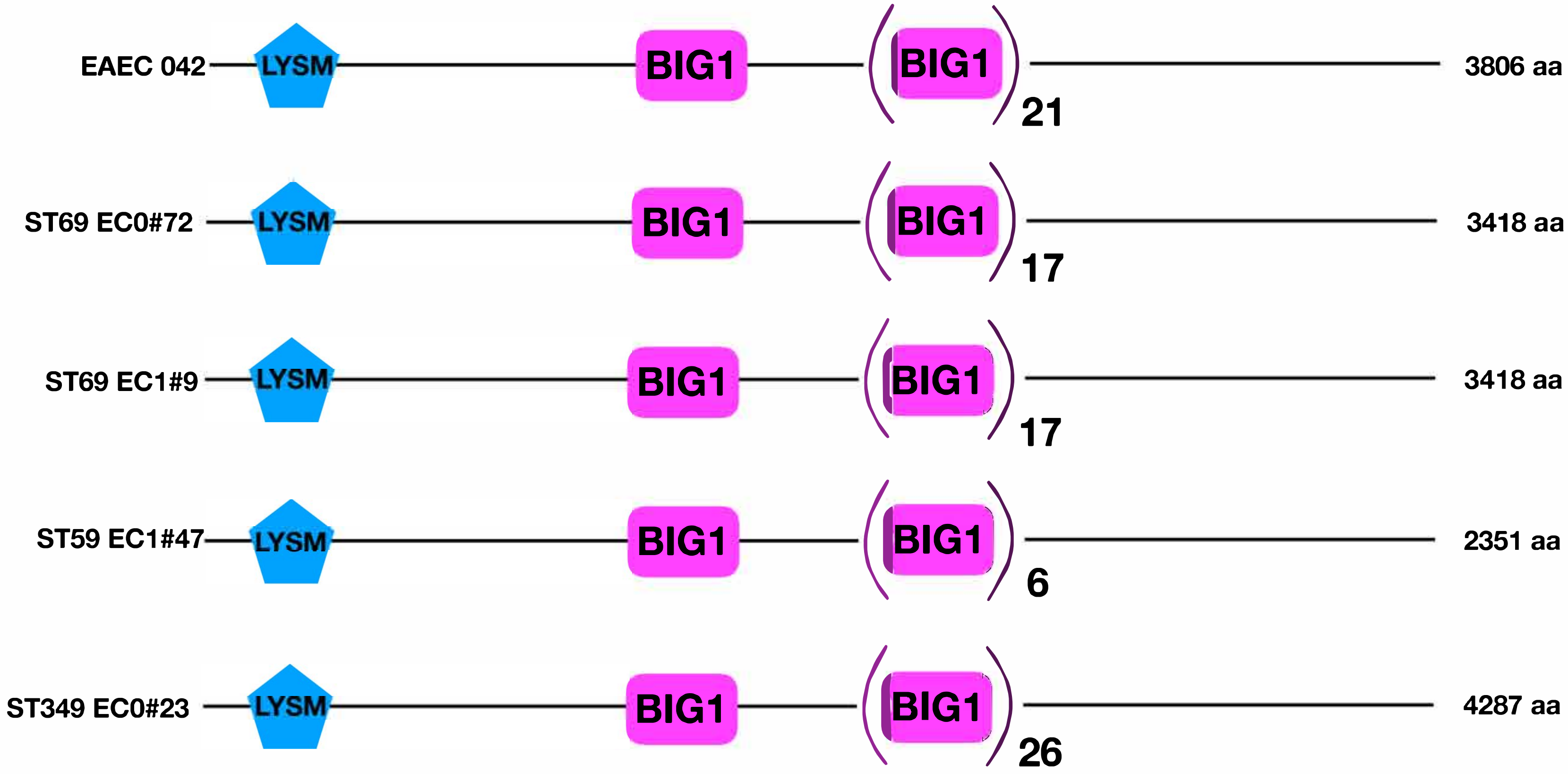

Figure S2. Bacterial Immunoglobulin-like repeats within the EaeX protein.
Domain structures were determined using Prosite and are shown for the indicated strains. LYSM is the LysM domain.

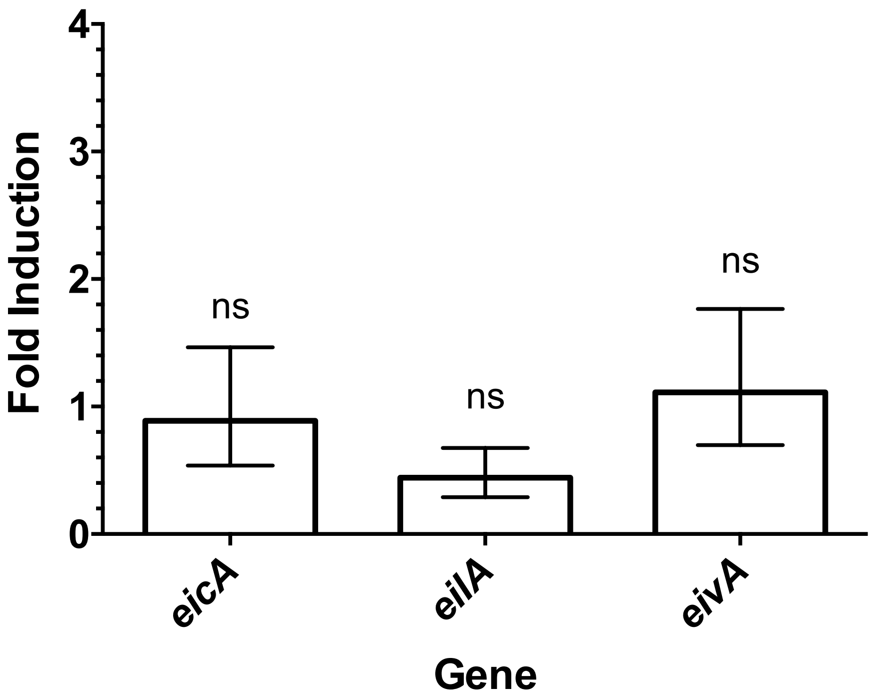

Figure S3. Expression of ETT2 genes assessed by quantitative PCR. Graph shows mean values of fold induction in LB:DMEM compared to LB alone for the indicated genes, normalised for expression of *gapA.* N=3 for each gene; error bars are ± sem. ns=not significant difference from 1.

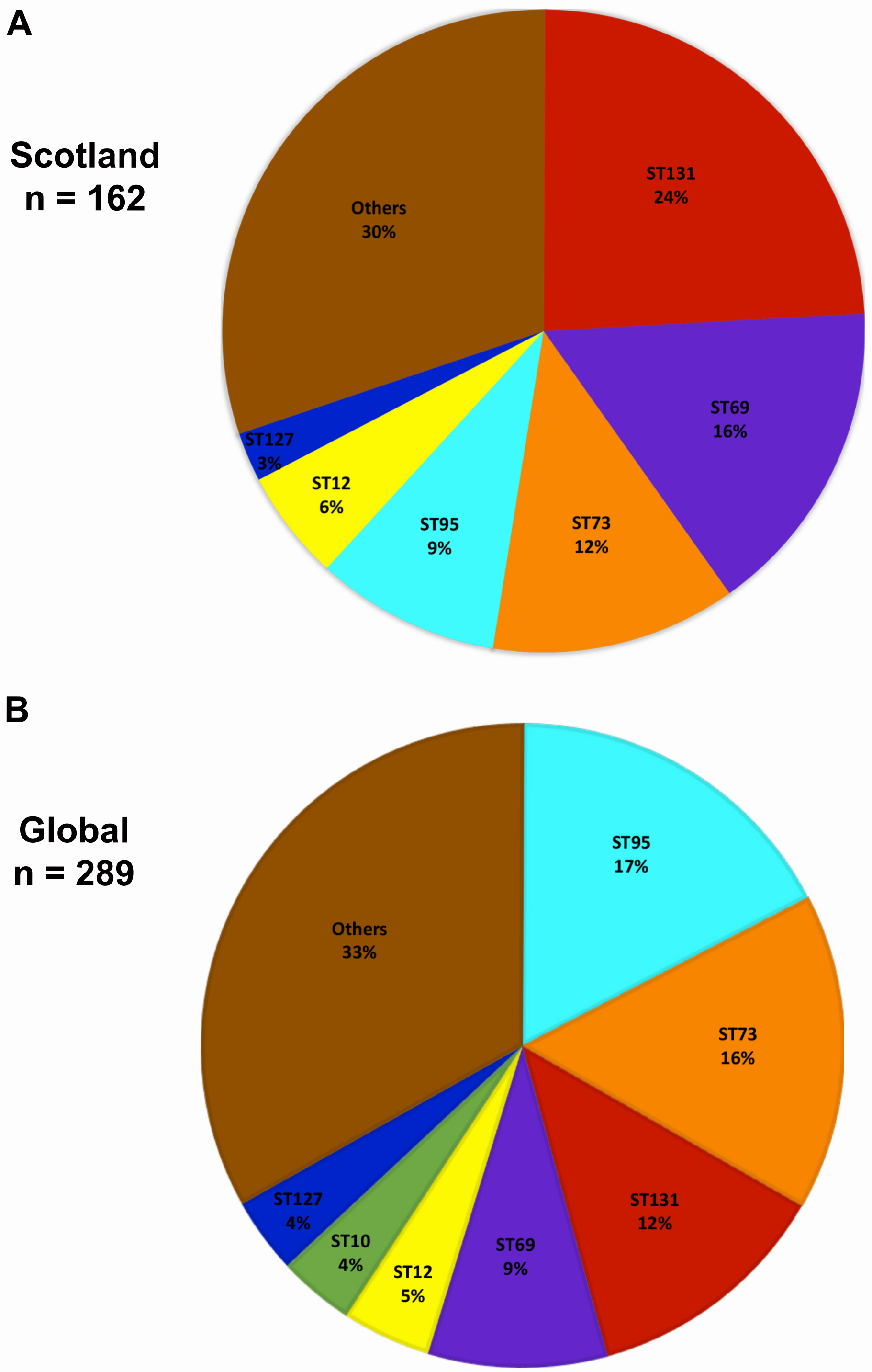

Figure S4. Distribution of STs among local blood stream isolates (A) and global samples (B). Total numbers of sequences analysed are shown by n =.

Gene	Bases	Sequence	%GC	Tm
eicA*(ygeG2)* forward	21	CAC TAG GTA TGG GAG CAA CAC	52.4	55.1
eicA*(ygeG2)* reverse	21	AGA GTT CAG CTT CGT CAA GTC	47.6	54.6
eilA*(ygeH_2)* forward	20	AAA GTC GAC CAC CAC TCT TG	50.0	54.8
eilA*(ygeH_2)* reverse	20	GCC CTG GAC AAG TTC GTA AT	50.0	55.0
eivA*(flhA_2)* forward	21	GCG TGA ATG GCG ATA GTG ATA	47.6	54.9
eivA*(flhA_2)* reverse	20	ACC TGG AAG CAA CCC AAT AC	50.0	55.1
gapA*_1* forward	21	CTG CTG AAG GCG AAA TGA AAG	47.6	54.6
gapA*_1* reverse	20	TAG CAT CGA ACA CGG AAG TG	50.0	54.9

Table S3. Primers used for quantitative PCR evaluation of the indicated genes.
